# Adjuvant therapy for T3N0 rectal cancer in the total mesorectal excision era- identification of the high risk patients

**DOI:** 10.1186/1748-717X-5-118

**Published:** 2010-12-15

**Authors:** Ji Zhu, Ye Xu, Weilie Gu, Junjie Peng, Gang Cai, Guoxiang Cai, Wenjie Sun, Weiqi Shen, Sanjun Cai, Zhen Zhang

**Affiliations:** 1 Departments of Radiation Oncology, Fudan University Shanghai Cancer Center, Shanghai, 200032, PR China; 2 Departments of Colorectal Cancer, Fudan University Shanghai Cancer Center, Shanghai, 200032, PR China; 3 Department of Oncology, Shanghai Medical College, Fudan University, Shanghai, 200032, PR China; 4 Departments of Pathology, Fudan University Shanghai Cancer Center, Shanghai, 200032, PR China

## Abstract

**Background:**

Adjuvant therapy for T3N0 rectal cancer was controversial with respect to both radiation and the use of a combined regimen of chemotherapy. We evaluated both clinical features and biomarkers and sought to determine risk factors for those patients retrospectively.

**Methods:**

A total of 122 patients with T3N0 rectal cancer were analyzed in this study from January 2000 to December 2005. Clinicopathologic and biomarkers were used to predict local recurrence (LR), disease-free survival (DFS), and overall survival (OS).

**Results:**

The median follow-up interval was 45.4 months. Five-year LR, DFS, and OS rates were 10.4%, 68.3%, and 88.7%. Having a lower tumor location and showing low P21 and high CD44v6 expression were identified as risk factors for LR: patients with two or three of these risk factors had a higher 5-year LR rate (19.3%) than did patients with none or one of these risk factors (6.8%) (p = 0.05). A poorer DFS was related to low P21 nor high CD44v6 expression but not to tumor location: the 5-year DFS rates were 79.3% for those with neither, 65.9% for those with either one or the other, and 16.9% for those with both (p = 0.00).

**Conclusions:**

The prognostic model including tumor location, P21 and CD44v6 expressions could help to distinguish these patients with high risk T3N0 patients and determine whether adjuvant therapy was beneficial.

## Introduction

Current guidelines from the National Comprehensive Cancer Network recommend that all patients with clinical stage II/III rectal cancer should be treated with preoperative chemoradiation followed by total mesorectal excision (TME). However, whether patients with T3N0 rectal cancer, i.e., those with tumors invades through the muscularis propria into perirectal fasia but no invasion of adjacent organs, and without lymph nodes invasion, should undergo such therapy is still controversial. It is believed that not all T3N0 patients but those with high risk patients should be treated. Clinical assessments of T and N status are mainly based on findings from clinical examination, supplemented by magnetic resonance imaging (MRI) and endoscopic ultrasonography (EUS). However, this approach has led to over- or under-staging of disease in approximately 20% of cases, leading to speculation that those patients may not have gotten optimal therapy [1,2]. Moreover, the effectiveness of neoadjuvant chemoradiation therapy in downstaging disease can cloud the accuracy of disease re-staging[3]-and T and N status are still considered the strongest predictors of outcome. The ability to predict which patients with T3N0 cancer are at the highest risk of recurrence would be useful for identifying which such patients are likely to derive the most benefit from adjuvant therapy and which patients may safely avoid such therapy, which is associated with adverse effects.

Some investigators have evaluated potential risk factors with the intent of identifying the best type of adjuvant therapy for rectal cancer, but unfortunately those studies were conducted before the advent of total mesorectal excision (TME) [4,5], which greatly reduces the risk of local relapse compared with conventional surgery [6]. Moreover, there is no prospective randomized trials to compare T3N0 rectal patients who had TME with adjuvant therapy or not. At present time, it is not ethical to conduct a prospective randomize clinical trial to investigate if adjuvant therapy is necessary in this special group of patients given the evidence of advantage of neoadjuvant over adjuvant chemoradiation in the whole stage II/III group of patients. Considering the existence of uncertainties either in the preoperative clinical staging or tumor downstaging after effective neoadjuvant chemoradiation may weaken the power of outcome driven from trials treated with current standard neoadjuvant strategy. Therefore, it is important to identify potential risk factors from patients with post-TME and with pathologically staged T3N0 disease to help determine the optimal treatment strategy for individual patients. The traditional clinical prognostic and risk factors have been reported but with no consensus reached so the combination with some biological factors would be more helpful. The biological or molecular markers combined with clinical factors to predict tumor prognosis have been applied in the guideline of breast cancer and may have potential value in the future staging system of colorectal cancer. Predictive value of biomarkers has been reported in our previous study, which demonstrated that CD44v6 expression in cancer cells was a sensitive marker for predicting treatment outcome in patients with stage II/III rectal cancer after TME [7].

To this end, we evaluated a variety of clinicopathological factors and molecular markers in patients with pathogically staged T3N0 rectal cancer after TME, with the goal of identifying factors related to predicting outcome.

## Materials and methods

### Patient selection

Subjects for this retrospective analysis were selected from 1306 patients with rectal cancer treated with TME at the Fudan University Cancer Hospital in Shanghai from January 2000 to December 2005. For the present analysis, the patient selection criteria were as the following: (1) without any preoperative therapy; (2) undergone TME surgery; (3) pathologically confirmed T3N0 rectal adenocarcinoma; (4) no evidence of distant metastases; (5) available to provide follow-up information at least once. After the medical and pathologic records were carefully reviewed, 131 patients with T3N0M0 were selected. Furthermore, only nine cases out of them received adjuvant radiotherapy because of beneficial uncertainty. Therefore, these 9 cases were excluded and a total of 122 patients were identified for our retrospective analysis.

### Immunohistochemical staining for biomarkers

All information on biomarker staining was extracted from the pathologic records. All dissected tumor specimens were routinely fixed in buffered formalin and embedded in paraffin. Sections of 4-mm thickness were stained with hematoxylin-and-eosin for histologic diagnosis and then labeled with six primary antibodies: anti-epidermal growth factor receptor (EGFR) (Clone H11, code M3563, DAKO; dilution 1:100), anti-P21 (Clone NCC-RAS-001, code M0637, DAKO; dilution, 1:100), anti-Her-2 (Clone: PN2A, code M7269, DAKO; dilution 1:400), anti-P53 (Clone: DO-7, code M7001, DAKO; dilution, 1:50), anti-CD44v6 (Clone: VFF-7, code M0130, Antibody Diagnostica Inc; dilution 1:50), and anti-proliferating cell nuclear antigen (PCNA) (Clone: PC 10, code M 0879, DAKO; dilution 1:300). For antigen retrieval, sections were treated with 10 mM citrate at pH 6.0 in a 750-W microwave oven for three 5-min cycles. The sections were then immunostained with horseradish peroxidase (HRP) polymer (DAKO) in accordance with the manufacturer's specifications. Diaminobenzidine was used to develop the stains and hematoxylin for the counterstaining. Negative controls consisted of substituting normal mouse serum for the primary antibodies. PCNA staining was expressed as a labeling index of the percentage of positively staining nuclei among all counted nuclei. Expression of the other five markers was quantified by using a visual grading system based on staining intensity on a scale from 0 to 3. These scores were routinely included in the pathology reports. For the purpose of this analysis, we collapsed the scores into two groupings: low intensity (0 or 1) or high intensity (2 or 3).

### Definition of treatment failure

All treatment failures were verified through review of the medical records. Local-regional failure was defined as recurrence within the pelvis, including the tumor bed, regional lymph nodes, anastomosis, or perineal scar. Distant failure was indicated as disease recurrence detected in the liver, lung, brain, and other organs outside the pelvis.

### Statistical analyses

The data used for this analysis included patient age and sex, distance of the tumor from the anal verge, maximum tumor diameter, cellular differentiation, the presence or absence of lymphatic or vascular invasion, the number of dissected lymph nodes, and whether adjuvant therapy had been administered. No patients got neoadjuvant therapy.

Six potential predictive molecular markers (EGFR, P21, Her-2, P53, CD44v6, and PCNA), detected by immunohistochemical staining of surgical specimens, were also evaluated as potential prognostic factors.

Characteristics were described in terms of frequency for the categorical variables, means and standard deviations for continuous data, and medians for non-normally distributed continuous data. Survival time was calculated from the date of surgery to the date of event or the last follow-up. Three events were defined: local-regional failure, disease-specific failure, and death. Data were censored from patients lost to follow-up, death from causes other than rectal cancer, or no event as of the last follow-up. Survival curves were estimated by using the Kaplan-Meier method and compared with log-rank tests in the univariate analysis. Cox proportional hazards regression was used for multivariate modeling and for examining the prognostic significance of the variables identified in the models. P values of less than 0.05 were taken to indicate statistically significant differences.

## Results

### Clinicopathologic characteristics

Patients' clinicopathologic characteristics are presented in Table 1. Sixty-six were men and 56 were women; the median age was 55 years (range, 27-88 years). Fifty patients (41.0%) had tumors location ≤ 5 cm from the anal verge, and 10 patients (8.2%) had lymphatic or vascular invasion. The median number of lymph nodes examined was 10 (range, 2-28). All patients had received 5-Fu based adjuvant chemotherapy. All patients had the detailed records of immunohistochemistry. The distribution of six molecular markers analyzed was listed in Table 2.

**Table 1:** The distribution of clinical pathological factors.

clinico-pathological factors	n
Gender	
Male	66
Female	56
Age (year)	
Mean ± Sd	57 ± 12
Surgery	
Lower anterior resection	60
Abdominal perineal resection	66
Distance from anal verge	
<= 5 cm	50
>5 cm	72
lymphatic or vascular invasion	
Yes	10
No	112
Resected Lymph node number	
Median (min-max)	10 (2-28)

Total	122

**Table 2:** The distribution of molecular markers.

Molecular markers	N
P21	
-	28
+	29
++	43
+++	22
CD44/v6	
-	72
+	27
++	20
+++	3
EGFR	
-	71
+	27
++	16
+++	8
Neu	
-	81
+	26
++	12
+++	3
P53	
-	45
+	27
++	27
+++	23
PCNA	
<= 50%	32
>50%	90

Total	122

**Figure 1:**
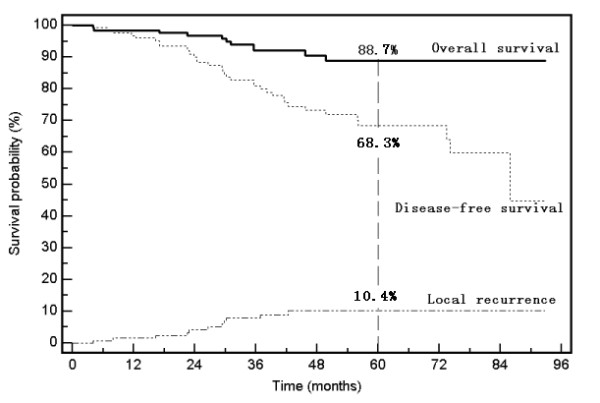
**Local recurrence, disease-free survival, and overall survival rates for all patients**.

The median follow-up interval was 45.4 months (range, 4.2-92.8 months). During the follow-up period, 11 patients developed recurrent disease within the pelvis, 25 patients developed distant metastases, and 9 patients died of rectal cancer. The 5-year LR, DFS, and OS rates for all patients were 10.4%, 68.3%, and 88.7%, respectively (Figure 1).

### Potential risk factors and prognostic significance

**Figure 2:**
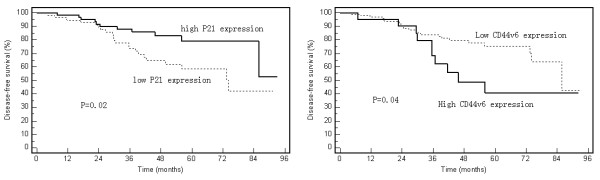
**Disease-free survival rates by P21 and CD44v6 expression levels**.

**Figure 3:**
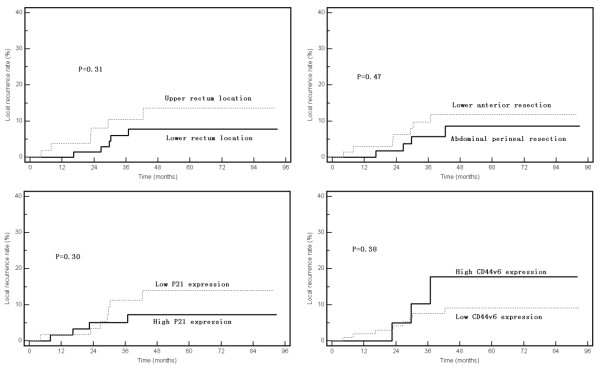
**Local recurrence rates by tumor location, type of surgery, and P21 and CD44v6 expression levels**.

All potential risk factors, including clinicopathologic factors and six molecular biomarkers, were evaluated by using the Kaplan-Meier method (compared with Log-rank test). Among six molecular biomarkers, only the expression level of P21 and CD44v6 exhibited a correlation with prognosis. Significant longer disease-free interval was displayed in patients with high expression of P21 (79.1% vs. 41.9%, p < 0.05) or low expression of CD44v6 (75.5% vs. 40.7%, p < 0.05) (Figure 2), and a tendency of less local relapse (P > 0.05) (Figure 3). For all clinicopathologic factors, the rectal lesion at a lower location (the distance from anal verge within 5 cm) and abdominoperineal resection exhibited a trend to an increased local failure rate compared with the upper lesion and low-anterior resection in spite of no statistically significance (Figure 3). None of the other variables displayed any correlative tendency with OS, DFS or LR (data not shown). As there wasn't any factor in our study showed correlation with OS, we only analyzed prognostic factors for LR and DFS.

### Cox multivariate regression models for predicting LR and DFS

**Figure 4:**
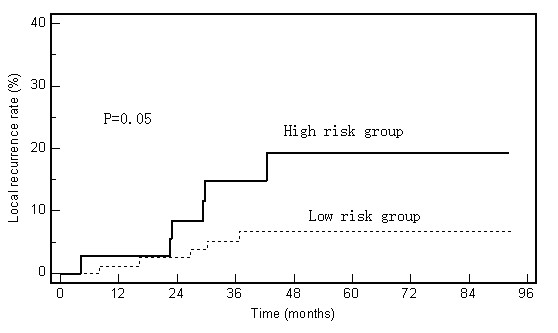
**Local recurrence rates according to the combined prognostic index (P21, CD44v6, and tumor location)**. High risk involves having two or three adverse risk factors (low tumor location, low P21 expression, and high CD44v6 expression). Low risk involves having no or one adverse risk factor.

Four variables-P21 expression level, CD44v6 expression level, tumor location, and type of surgery-showed effects on LR (Figure 2). Due to the tumor location and type of surgery were highly correlated with each other (*r *= 0.71, p = 0.00), the type of surgery was excluded from further analysis. Cox multivariate regression analysis revealed three factors to be associated with LR: P21, CD44v6, and tumor location (Table 3). Because the hazard ratios of these three factors were very close to one another, we combined these three factors into a prognostic scoring system, with each of the three unfavorable prognostic factors allocated 1 point. We then assigned the patients to one of two risk-of-LR groups according to their score in this system: low risk (score of 0 or 1) or high risk (scores of 2 or 3). The 5-year LR rates were significantly different for the low-risk group (6.8%) and the high-risk group (19.3%) (p = 0.05, Figure 4).

**Table 3:** the values of β and P in Cox multi-variate regression model for local recurrence and disease-free survival.

		β	hazard ratio	P value
Local recurrence	P21 (high:low)	0.591	1.806	0.35
	CD44/v6 (low:high)	0.628	1.874	0.36
	Tumor location (upper:lower)	0.593	1.809	0.33
Disease-free survival	P21 (high:low)	0.925	2.522	0.01
	CD44/v6 (low:high)	0.842	2.321	0.03

**Figure 5:**
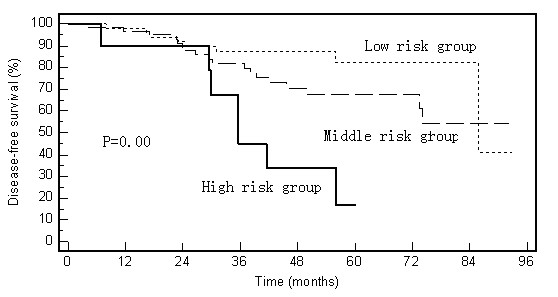
**Disease-free survival rates according to the combined biomarker prognostic index (P21 and CD44v6)**. High risk involves having both of the two risk factors: low P21 expression and high CD44v6 expression. Intermediate risk involves having either low P21 expression or high CD44v6 expression. Low risk involves having neither low P21 expression nor high CD44v6 expression.

Different from the LR, tumor location is not the risk factor of DFS (Table 3). We derived a separate scoring system for the risk of DFS that was based on only two variables-expression of P21 and CD44v6. Similar scoring system as used in LR were generated for analyzing DFS: a score of "0" was defined as high P21 or low CD44v6 expression, and "1" was low P21 or high CD44v6. Use of that scoring system led to the identification of three risk groups: low risk (score of 0), intermediate risk (score of 1), and high risk (score of 2). The 5-year DFS rates for these three risk groups were significantly different: 79.3% for the low-risk group, 65.9% for the intermediate-risk group, and 16.9% for the high-risk group (p = 0.00, Figure 5).

## Discussion

Our analysis of potential predictors of outcome after TME for T3N0 rectal cancer revealed that three factors were associated with a higher risk of LR: the tumor being located close (≤ 5 cm) to the anal verge, expressing low amounts of P21, and expressing high amounts of CD44v6. Patients with no or only one risk factor had a relatively low risk of LR at 5 years (6.8%). In contrast, the probability of LR in 5 years is high up to nearly 20% when two or three risk factors were presents in the high-risk group patients. Thus adjuvant radiotherapy should be considered for those patients at high risk of LR according to our scoring system, but 93.2% of those with low-risk disease may benefit from sparing of treatment induced toxicity with post-operative radiation.

Other than risk factors with LR, we further found that two factors were associated with the risk of decreased DFS: low P21 expression and high CD44v6 expression. These factors may also be useful in deciding whether a particular patient should undergo adjuvant therapy or not. However, further testing of much larger groups of patients and additional potential prognostic factors are needed to definitively establish the need, or the role, of adjuvant therapy for these patients.

As a subgroup of stage II rectal cancer, T3N0M0 represent a different response to clinical treatment. Gunderson and his colleagues pooled data from five pre-TME era phase III randomized trials of adjuvant therapy for rectal cancer and found similar OS and DFS rates among patients with T3N0 cancer treated with adjuvant chemotherapy or chemoradiation [8]. These findings raised concern that trimodality therapy may have represented overtreatment for some of these patients.

Other investigators have attempted to identify risk factors related to outcome for patients with T3N0 rectal cancer who undergo a conventional radical surgery but TME procedure was not routinely administrated in their reports. Clinical factors tested for prognostic value have included cell differentiation, tumor location, number of examined lymph nodes, vascular/lymphatic invasion, and circumferential resection margin status. In one such analysis of 95 patients with T3N0 rectal cancer at the Memorial Sloan-Kettering Cancer Center, Merchant et al. reported that only lymphatic invasion was significant to predict LR [5]. In another report by Willett et al., histologic type of well to moderately differentiated, tumor invasion of < 2 mm into the perirectal fat, and without lymphatic or venous vessel involvement were shown to be favorable histologic features [4]. Other risk factors, such as lower rectal tumor location and inadequate lymph node dissection, have also been reported [9,10]. However, there are two issues we have to face: firstly, all these conclusions are drawn from pre-TME studies; secondly, there are no consistent risk factors confirmed in these studies. Therefore, these conclusions can't be applied arbitrarily now in TME era.

With appearance of TME surgery, local control and survival rates have been improved greatly [6]. Several trials have reported that TME surgery alone can reduce the local failure rates from 15%-20% to 4%-7% and improve the survival rate to 80%-85% for patients with stage II disease [11-14]. Given that patients with more advanced disease were excluded from these early trials of TME, the excellent outcome can be largely attributed to patient selection bias. But, due to the excellent local control with TME, it also raises the concern of overtreatment in this intermediate risk group, especially in T3N0 patients. In our hospital, TME has been routinely used for rectal cancer patients more than 10 years. It is the optimal solution to select patients with high risk factor to be treated as to avoid the possibility of overtreatment after TME. However, no concurrent data from randomized trials are available to support the omission of adjuvant therapy. Our scoring system will be helpful in treatment decision for T3N0 patients.

On the other hand, apart from clinical characteristics, the inconsistent conclusion may be partly attributable to differences in the biological behavior of tumors determined by intrinsically different molecular regulatory mechanisms. In our study, a scoring system based on CD44v6 and P21 expression showed significant prognostic value for both LR and DFS in T3N0 group of patients which may help to decide treatment biologically.

CD44v6 is a cell membrane glycoprotein involved in cell/cell and cell/matrix interactions. Over-expression of CD44v6 correlates with tumor growth and spread. Some studies have found increased levels of CD44v6 in tumors compared with expression in normal tissues [15]. High CD44v6 expression has been associated with metastatic disease and a poor prognosis in colorectal cancer [16-18].

It has also been reported that P21, through interaction with cyclin-dependent kinases, can induce cell cycle arrest at the G_1_/S phase, where cells are insensitive to radiation-induced DNA damage [19]. In cell line models, the prognostic value of P21 expression has also been reported in colon cancer cells [20,21]. Fu et al. speculated that rectal tumors that express P21 expressions may be more radiosensitive to preoperative radiation therapy and thus be associated with better prognosis [22]. Similar results were reported by Havenga et al. [14]. In another clinical research report, Rau et al. found that increased P21 expression was associated with decreased lower proliferative activity after neoadjuvant chemoradiation[23].

Based on our data showed, we find some clinical risk factors as well as biomarker to predict the outcome and the generated scoring system may help to provide more tailored therapy in T3N0 patients. Based on the scoring system that we used, the 5-year LR varied from 19.3% to 6.8% according to risk grouping, suggesting adjuvant radiation may be benefit in high risk patients with at least two of the three identified risk factors (low tumor location, low P21 expression, and high CD44v6 expression) in decreasing LR.

Our scoring system is also helpful for evaluation the outcome of DFS which may be more associated with adjuvant chemotherapy other than radiation. In terms of adjuvant chemotherapy, the regimen for rectal cancer was extrapolated from that for colon cancer even though there is no definitive evidence to clarify they are the same disease. Chemotherapy is also indicated in high risk stage II patients and the chemotherapy regimen in stage II patients is not as uniform as with stage III patients. In stage II colon cancer, the MOSIC randomized phase III trial has showed the similar results of 5-year DFS rates with intensive (FOLFOX) to conventional (5-FU/LV) chemotherapy regimen (83.7% vs 79.9% p = 0.258), suggesting that the possibility of sparing more intensive treatment in stage II colon cancer patients. There is no data to illustrate if more aggressive treatment will be benefit when result of stage II colon cancer derived into rectal cancer with same staging. Our scoring system showed significant difference of 5-year DFS of 16.9% to 79.3% with high and low score T3N0 patients and suggested a more aggressive chemotherapy may be appropriate for this group of rectal cancer patients with high score.

Therefore, we conclude that the scoring system we developed in this study may be useful for identifying whether patients could be benefit from adjuvant therapy after TME for T3N0 rectal cancer or not. On the basis of our results, we suggest that adjuvant radiotherapy is required for high-risk patients, and that a more aggressive chemotherapy regimen should be recommended in the patients with increased risk scores. Molecular markers tested in the pre-treatment biopsy tissue may be helpful for selecting patients with potential high risk of recurrence. Such study has been reported in 30 patients from German CAO/ARO/AIO-94 rectal cancer study. There were 54 genes found to be different between responder and non-responders of neoadjuvant chemoradiation in 23 patients. This may suggest the potential possibility in decision making with molecular markers combined pretreatment[24]. However, this strategy has been in the status of exploration and there is no consensus reached for specific gene expression so far. We have undergoing a perspective study in locally advance rectal cancer patients which is exploring the molecular markers which may be radiosensitive and radioresistant to select patients more suitable for neoadjuvant chemoradiation. So, we will combine clinical features and molecular markers in our ongoing study. In the future, further prospective randomized trials will be focused on investigating the optimal treatment strategy in stage II (T3N0) rectal cancer. Our scoring system will provide a basic reference as well as pretreatment molecular markers for future study in this group of patients.

## Conclusions

In conclusion, we propose that the scoring system including tumor location, P21 and CD44v6 expressions could help to distinguish these patients with high risk T3N0 patients and determine whether adjuvant therapy was beneficial. The optimal treatment strategy in T3N0 rectal cancer will be investigated in the future studies.

## Competing interests

The authors declare that they have no competing interests. They also declare no financial disclosure.

## Authors' contributions

ZJ and XY conceived and drafted the manuscript, ZZ drafted and revised the manuscript, and all authors read and approved the final manuscript.
